# The evidence base of interventions to treat antenatal depression: a meta-analysis of randomized controlled trials

**DOI:** 10.1007/s00737-026-01723-0

**Published:** 2026-07-03

**Authors:** Riddhi Laijawala, Kristi Priestley, Rebecca Bind, Katie Hazelgrove, Lavinia Rebecchini, Madeline Kirkpatrick, Francesca Mancino, Samrina Sangha, Alessandra Biaggi, Malvika Godara, Claudia Buss, Sonja Entringer, Anthony Woods, Paola Dazzan, Annamaria Cattaneo, Carmine M Pariante

**Affiliations:** 1 Department of Psychological Medicine, Institute of Psychiatry, Psychology and Neuroscience, Kings College London, London, United Kingdom; 2 Department of Pharmacological and Biomolecular Sciences, University of Milan, Milan, Italy; 3 Institute of Medical Psychology, Charité—Universitätsmedizin Berlin, Berlin, Germany; 4 German Center for Child and Adolescent Health, Berlin, Germany; 5 German Center for Mental Health, Berlin, Germany; 6 Department of Psychosis Studies, Institute of Psychiatry, Psychology and Neuroscience, Kings College London, London, United Kingdom; 7 Biological Psychiatry Unit, IRCCS Istituto Centro San Giovanni di Dio Fatebenefratelli, Brescia, Italy

**Keywords:** Antenatal depression, Randomised controlled trials, Interventions, Meta-analysis

## Abstract

**Purpose:**

Antenatal depression (AD) is a mental health condition estimated to affect close to 30% of pregnant people globally. The importance of treating depression in pregnancy is underscored by its downstream impact on both maternal and infant biopsychosocial outcomes, such as preterm birth, low birthweight, and postnatal depression. Despite these outcomes, only 20% of women experiencing AD receive appropriate and timely support and treatment. This meta-analysis is a comprehensive synthesis of RCTs evaluating psychological and non-psychological interventions to treat antenatal depression, conducted as part of the HappyMums project.

**Methods:**

Studies were sourced from PubMed, Embase Medline and MIDIRS (via OVID), and were included if they were (a) randomized controlled trials, (b) with pregnant people with, or at risk for depression, (c) consisted of an intervention, and (d) published in English. To estimate efficacy, Standardized Mean Differences (SMDS) were calculated with 95% confidence intervals using the post- intervention Ms and SDs of both the intervention and control groups. The I^2^ statistic was used as an indicator of variation between studies. Analyses were conducted using the software R Studio.

**Results:**

In total, 115 studies were included in the analysis. The pooled effect size of all interventions, compared with all pooled control arms, showed a clear therapeutic effect (SMD = 0.65, 95% CI 0.48; 0.83), although the between-study heterogeneity variance was high (τ^2^ = 0.78, 95% CI 0.7;1.3). After accounting for outliers, the pooled effect size across 94 treatment arms was still SMD = 0.5 (95% CI, 0.42, 0.56). No significant subgroup differences were found by intervention type and format, or measure used.

**Conclusion:**

There are numerous interventions that, on average, are moderately effective in reducing depressive symptomology in pregnancy. Future research should be aimed at addressing how to personalise the choice of treatment and improve treatment outcomes for the individual pregnant person.

**Supplementary Information:**

The online version contains supplementary material available at https://doi.org/10.1007/s00737-026-01723-0.

## Introduction

Antenatal depression (AD), also known as prenatal depression, is a mental health condition estimated to affect 28.5% of pregnant people globally (Al-Abri et al. 2023). While there are many evidence-based treatment pathways available, what remains largely unknown is the comparative efficacy of these interventions, and this meta-analysis synthesizes such evidence. The importance of treating depression in pregnancy is underscored by its adverse impact on both maternal as well as offspring outcomes. These include adverse birth outcomes, such as preterm delivery (Jarde et al. 2016; Smith et al. 2020; Yedid Sion et al. 2016) and low birthweight (Field 2017; Jarde et al. 2016) and adverse psychosocial offspring outcomes, like insecure or disorganised attachment (Hayes et al. 2013) and temperamental and sleep-related disturbances (Smith et al. 2020). Indeed, the literature also portrays AD as among the most prominent risk factors in the development of postpartum depression (Leigh and Milgrom 2008). Despite these outcomes, only 20% of women experiencing AD receive the appropriate intervention (Vigod et al. 2016), with factors such as lack of access, a long waitlist for treatment, and prior negative experience with mental healthcare, posing as barriers to accessing treatment (Kopelman et al. 2008).

Existing systematic reviews and meta-analyses have demonstrated the efficacy of CBT (Shortis et al. 2020), mindfulness-based interventions (Li et al. 2022), lifestyle and exercise-based interventions (Zhang et al. 2024) in the treatment of antenatal depression. Indeed, with the advancement of digital health tools within the space of perinatal mental health, evidence has shown that digital therapeutic interventions are also effective tools in managing depressive symptomology during pregnancy (Lau et al. 2021). However, to our knowledge, no meta-analyses have synthesized the entire evidence base, encompassing psychological, non-psychological, as well as digital interventions in the treatment of antenatal depression using a common methodological approach and effect sizes calculation; therefore, this was our primary aim. Furthermore, we included an analysis of biomarkers changes during the interventions, in the smaller set of studies that have this data, to provide a mechanistic insight into how these diverse interventions might work.

While meta-analyses have been undertaken on specific intervention types, such as exercise (Zhang et al. 2024) and psychotherapy (Li et al. 2020), no meta-analysis at the time of data extraction had provided an overarching synthesis of the evidence base, considering all the available treatment pathways. In the absence of any randomised controlled trials using a pharmacological intervention (Howland 2013), we felt it was important to establish the range of psychological and non-psychological interventions that were effective, and then we followed-up this global investigation by undertaking subsequent subgroup analyses by intervention format and delivery type.

An increasing amount of literature highlights the role of specific biomarkers, such as abnormal proinflammatory cytokines (for example, IL-6, IL-8, CRP, TNF alpha, IL-7, IL-10) (Silva-Fernandes et al. 2024; Zuo et al. 2025), and dysregulated HPA axis functioning (Carnevali and Buoli 2021), in the development of antenatal depression. Given this evidence, we deemed it appropriate to consider the impact that interventions have not only on depression, but on biological changes that might be causally related to the depressive symptoms and/or involved in the mechanisms of action of the interventions. 

Prior syntheses of the literature (Zeng et al. 2025) have focused on non-pharmacological interventions in the treatment of antenatal depression, however, to date no meta-analysis has synthesized the overall evidence base assessing depression, while also examining a range of biomarkers relevant to the perinatal period. Numerous biomarkers relevant to major depressive disorder have also emerged as prominent in perinatal depression treatment response, such as TNF alpha, IL-6, IL-10 (Osborne et al. 2018) and altered metabolites such as amino acids and the tryptophan-kynurenine pathway (Kwok et al. 2025). Therefore, we build on this knowledge base by exploring the evidence base of intervention studies which also include biomarkers. We initially aimed to investigate inflammatory biomarkers due to pregnancy being so clearly associated with the immune system. However, our review of the literature found varied markers (not only inflammatory), such as cholesterol, cortisol, and glucose. Above all, we integrated clinical data along with biomarkers so that we could have a more personalized understanding of treatment mechanisms, allowing for more tailored intervention strategies based on individual biomarkers’ profiles (Plant and Barton 2020). Since pregnancy is a time of great biological changes, it is vital to understand how effective interventions act from a biopsychosocial lens.

To mitigate the impact of antenatal depression on maternal and offspring outcomes, it is vital to review the evidence base of interventions. Therefore, as part of the HappyMums interdisciplinary research programme (funded by Horizon research and innovation) this meta-analysis was conducted to evaluate the effectiveness of all interventions for AD, both psychological and non-psychological (for example, lifestyle or alternative medicine), to gain an understanding of their comparative efficacy and the factors predicting the response. Overall, the effects size differences, albeit statistically significant, were small, and indicative of small changes in clinical scores which could be of doubtful clinical significance, for example approximately 2.3 points on the EPDS (Lautarescu et al. 2022), 1.6 points on the PHQ-9 (Srisurapanont et al. 2023) and 4.4 points on the CES-D (Kliem et al. 2020).

We also simultaneously identify, when available, biomarkers that are influenced by the interventions, to provide possible mechanistic insight into the therapeutic action.

## Methods

We followed the Preferred Reporting Items for Systematic Reviews and Meta-Analyses (PRISMA 2020) guidelines. Embase, PsycINFO, Medline, and the Maternity and Infant Care Database (MIDIRS) were searched via Ovid and utilised to extract relevant studies. Search terms included a combination of the following keywords: (pregnan* or prenatal or antenatal), AND (treatment or therapy or intervention) AND (depress*) to identify publications relevant to the research question. At this stage, a preferred study methodology was not included in eligibility criteria; however, in the subsequent screening we only selected randomised controlled trials. The search was conducted in successive waves from the beginning of HappyMums (1st of November 2022) until 30th of November 2024; although the review per se was not registered at inception, we followed the overarching aims of HappyMums as defined in the protocol (Biaggi et al. 2025) as well as the objective of the Project Task 3.5 (to conduct meta-analytic pooled estimates assessing the response to these interventions over depressive symptomatology, as wells as possible associations between the clinical improvement and biological factors). All identified studies were exported to Zotero Version 7.0.311, where duplicates were removed prior to the screening of abstracts and titles. To determine study eligibility, all studies were screened in a two-stage process: (1) A preliminary abstract and title screening to deem initial eligibility. In this, studies were categorised into “Eligible”, “Ineligible”, and “Maybe” categories. For studies where the primary reviewer (R.L.) was uncertain about eligibility (i.e. the “Maybe” group), a senior postdoctoral member of the team (K.P.) reviewed these studies and determined final eligibility. (2) In Stage 2, a full text review of “Eligible” studies was completed to identify the final studies for data extraction. At this stage, only randomized controlled trials (RCTs) were deemed eligible, as they are the ‘gold standard’ for effectiveness research (Hariton and Locascio 2018).

Studies were included in Stage 1 if they (a) Contained a sample of pregnant women (b) Assessed depressive symptomology as an outcome measure, either through self-report or clinician rated measures, (c) Delivered an intervention for antenatal depression, (d) Had a control condition such as treatment as usual or other active treatment and (e) Published in the English language. During Stage 2, RCT study methodology was introduced as an additional criterion.

Exclusion criteria were as follows (a) Studies not published in English (b) Dissertations, book chapters or conference abstracts (c) Non-randomised controlled trials. 

### Data extraction

Data was extracted from papers meeting eligibility criteria during Stage 2 of selection. The following data was extracted and entered in a table on an Excel file: Author, date of publication, country of delivery, gestational age at baseline, mean age of participants at baseline, intervention details, control condition details, study methodology, number of participants in each group, outcome measure used, outcome measure type (clinician or self-rated), Time 1 and Time 2 Means (M) and Standard Deviations (SD). Where studies consisted of more than two intervention arms, each treatment arm was compared to the arm pre-specified as a control group by study authors. In situations where necessary data for analysis was unavailable, corresponding authors were contacted. If no necessary data was available, the study was then excluded at the final stage before statistical analysis.

Data extraction was performed by R.L., to limit variability in the calculation of effect sizes. However, the data were re-examined by doubled entry by another member of the team (F.M.) before effect sizes were calculated. Moreover, for studies where the primary reviewer (R.L.) was uncertain about eligibility (i.e. the “Maybe” group), a senior postdoctoral member of the team (K.P.) reviewed these studies and determined final eligibility. Considering the classification of studies into specific subgroups, R.L. initially completed the classification, and M.K. reviewed these, helping confirm subgroup characteristics. Therefore, at each stage, the data was looked at by two independent researchers.

### Statistical analysis

#### Effect size calculations

To estimate efficacy, Standardized Mean Differences (SMDS), also known as Cohen’s *d* were calculated with 95% confidence intervals using the post-intervention Ms and SDs of both the intervention and control groups. This method of estimating effect sizes was chosen based on literature highlighting that within group (or pre-post) SMDs can be potentially contribute to biased outcomes and might not provide reliable information about intervention effects (Cuijpers et al. 2016).

All calculations were conducted on R, using pre-developed scripts (Harrer et al. 2021). To facilitate interpretation, the following guidelines recommended by Cohen were used: SMD 0.2 = Small, SMD = 0.5 Medium, SMD = 0.8 Large (Cohen 2013; Faraone 2008).

#### Publication bias

Publication bias was assessed by examining funnel plots of the included studies, and Egger’s tests as well as Duval and Tweedie’s trim and fill method (Duval and Tweedie 2000; Harrer et al. 2021)were utilised to examine plot asymmetry. Additionally, a risk of bias assessment was undertaken using the RoB 2 tool ((*RoB 2: A Revised Tool for Assessing Risk of Bias in Randomised Trials | The BMJ*, 2019.). R.L. completed the risk of bias screening, with additional support from S.S. Both used the guiding statement from the RoB tool for each domain in determining overall risk of bias in each study. If disagreements arose, R.L. and S.S. had a discussion to reach consensus and determine final risk of bias assignment.

#### Heterogeneity

The I^2^ statistic was used as an indicator of variation between studies. 25% heterogeneity was considered low, 50% moderate, and 75% was considered high (Melsen et al. 2014).

## Results

### Characteristics of included studies

**Fig. 1:**
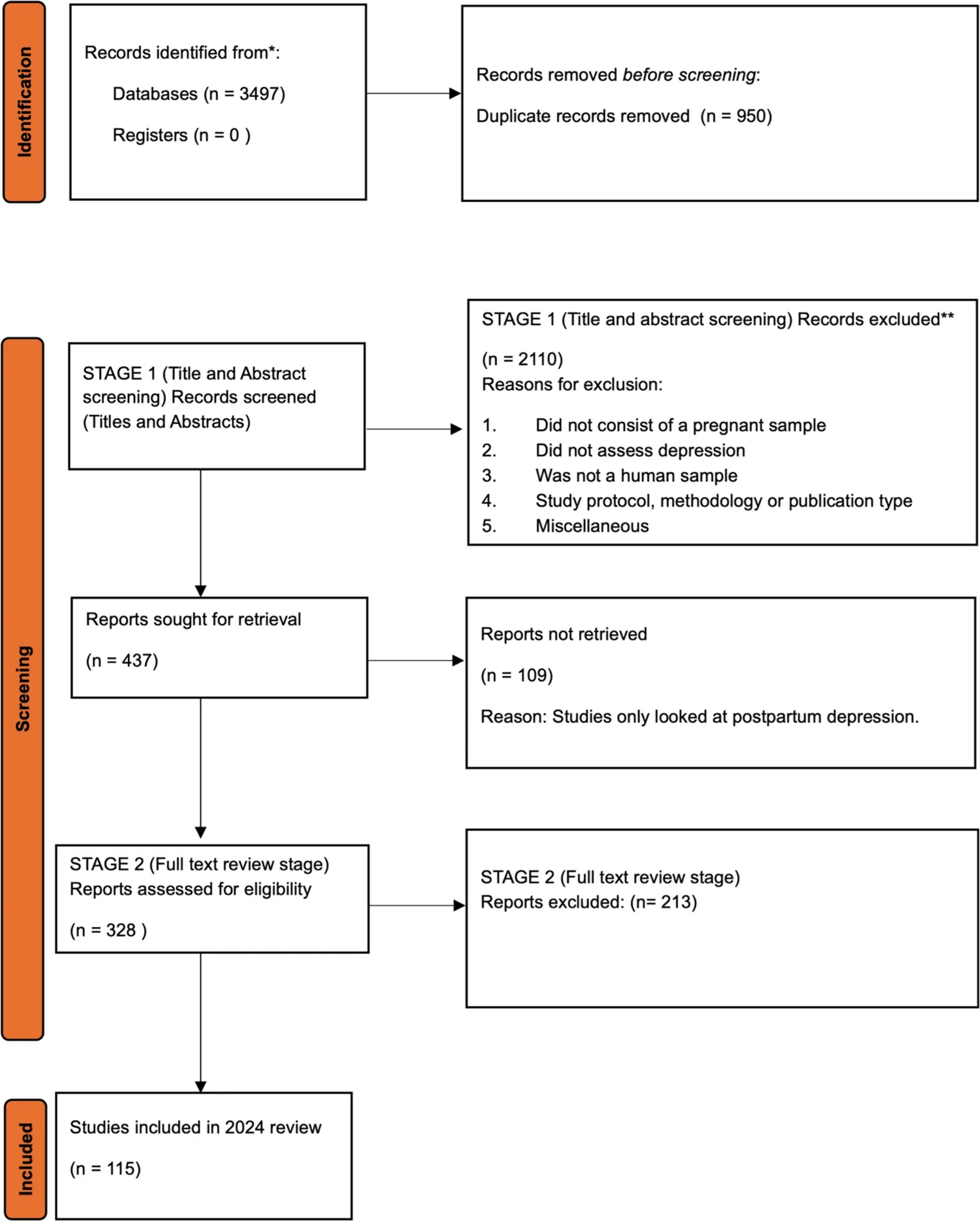
PRISMA 2020 flow diagram of study selection process, divided into (**a**) Identification stage, (**b**) Screening stage, and (**c**) Included stage

After the removal of 950 studies (which were duplicates), 2547 titles and abstracts were screened to deem initial eligibility. Out of 437 full text articles (deemed eligible at title/abstract screening), 328 met eligibility criteria. Finally, 115 trials (125 arms overall) were included in the meta-analysis of interventions. The study selection process is summarised in Fig. 1.

78 trials used standard antenatal care as a control group, 7 trials utilised a placebo condition, 2 used a sham control, 24 had an active control condition, and 4 trials did not specify details about the control group.

For the purpose of the analysis, intervention types were divided into (a) Psychological (68 studies), (b) Non psychological (43 studies), and (c) Combination (4 studies (see below for details)). 76 interventions were conducted in person, 27 were digitally/online based, 9 were conducted through monitored self-administration, and 3 studies utilised a combination of these intervention formats.

### Participant and regional characteristics

Overall, the trials included a sample of 12,561 participants, 6279 from treatment groups and 6282 from control groups. Accounting for missing values, the mean age of participants across all trials was 29.7 (SD 2.68), and the average gestational age at baseline was 21.8 weeks (SD 5.7).

Supplementary Material Table 1 summarises the geographical and income regions where the various trials were conducted. For consistency, The World by Income and Region (2024) of the world by income and geographical region were utilised. A similar number of studies were from high income countries (The World by Income and Region, *n* = 59) and from low to upper-middle income countries (*n* = 56). More details are in Supplementary Material Table 1.

### Effectiveness of interventions

Outliers were removed in an exploratory manner, once results were available on the substantial levels of heterogeneity and on the presence of publication bias as identified by the Egger’s test. This was done through the package ‘demetar’ on R studio (Harrer et al. 2021), whereby a removal algorithm searches for outlying studies from the initial meta-analysis, identifies them (with the option to remove and recalculate results). Once these outliers were identified, we subsequently removed them and then conducted the Egger’s test again. When 31 outliers were removed, the pooled effect size from 94 treatment arms was still indicative of a therapeutic effect (SMD = 0.5, 95% CI 0.42; 0.56, *p* < 0.001). Between study heterogeneity variance improved, as estimated at τ^2^ = 0.05 (95% CI 0.02; 0.09), with I^2^ value of 46.3% (95% CI 31.5%; 57.9%), and there was no longer any publication bias (see also below). Upon removing outliers, the 95% prediction interval was 0.06–0.92, this time clearly indicating that future studies with these interventions would yield therapeutic benefit in the treatment of antenatal depression.

#### Psychological interventions

Interventions were classified as psychological if they primarily targeted emotional, cognitive, or interpersonal processes, and non-psychological if they focused on physical, nutritional, lifestyle, or sensory strategies.

Psychological treatments included Mindfulness Based Interventions (MBIs), Cognitive Behavioural Therapy (CBT), psychoeducation, Interpersonal Psychotherapy (IPT), behavioural management, problem solving therapy, mentalization, counselling, music therapy, psychosocial intervention, cognitive analytic therapy, emotional management programmes, and behavioural activation.

#### Non psychological interventions

The range of non-psychological intervention types was large. Trials utilised interventions such as aromatherapy, religious and spiritually based treatments, bright light therapy, transcranial direct current stimulation, transcranial magnetic stimulation, yoga and exercise, Omega 3 supplementation, acupuncture, massage therapy, and childbirth education. Surprisingly, we could not find any randomised controlled trial of a pharmaceutical agent in AD.

#### Combination treatments

Interventions were categorised into the combination group if they integrated both psychological and non-psychological aspects into the interventions. Examples include interventions consisting of counselling sessions along with lifestyle-based couple training, and exercise coupled with cognitive analytic therapy.

### Measures used to assess depression

Included studies used a range of self-report and clinician-rated measures, specific to perinatal depression or for the general population. The most used measure was the Edinburgh Postnatal Depression Scale (EPDS) (Cox et al. 1987); while this measure uses the term postnatal, it has indeed been validated for use during pregnancy (Bergink et al. 2011). Other self-report measures included the Beck Depression Inventory (BDI) (Beck et al. 2011), Center for Epidemiological Studies-Depression Scale (CESD) (Radloff 1977), and the Patient Health Questionnaire 9 Item (PHQ-9) (Kroenke et al. 2001). Fewer studies reported on depression assessed using clinician-rated measures, such as the Hamilton Depression Rating Scale (HAM-D) (Hamilton 1960)and the Montgomery-Åsberg Depression Rating Scale (MADRS) (Montgomery and Åsberg 1979).

### Intervention format

Along with varied intervention types, the format of delivery also varied between the trials. 76 trials were conducted in person, that is, in a physical setting, where the participants were face to face with the facilitator. In person interventions were either conducted individually, or in a group. 27 digital/online interventions were delivered through a variety of means, such as smartphone apps, messaging services, Compact Discs (CDs), and via telephone. 9 studies used a self-administrated form of treatment, such as omega 3 supplementation, bright light therapy, self-help treatment, lavender cream treatment, and aromatherapy. Finally, 3 studies used a combination format, integrating online and in person formats.

### Differences in efficacy by intervention type, format and measure

**Fig. 2:**
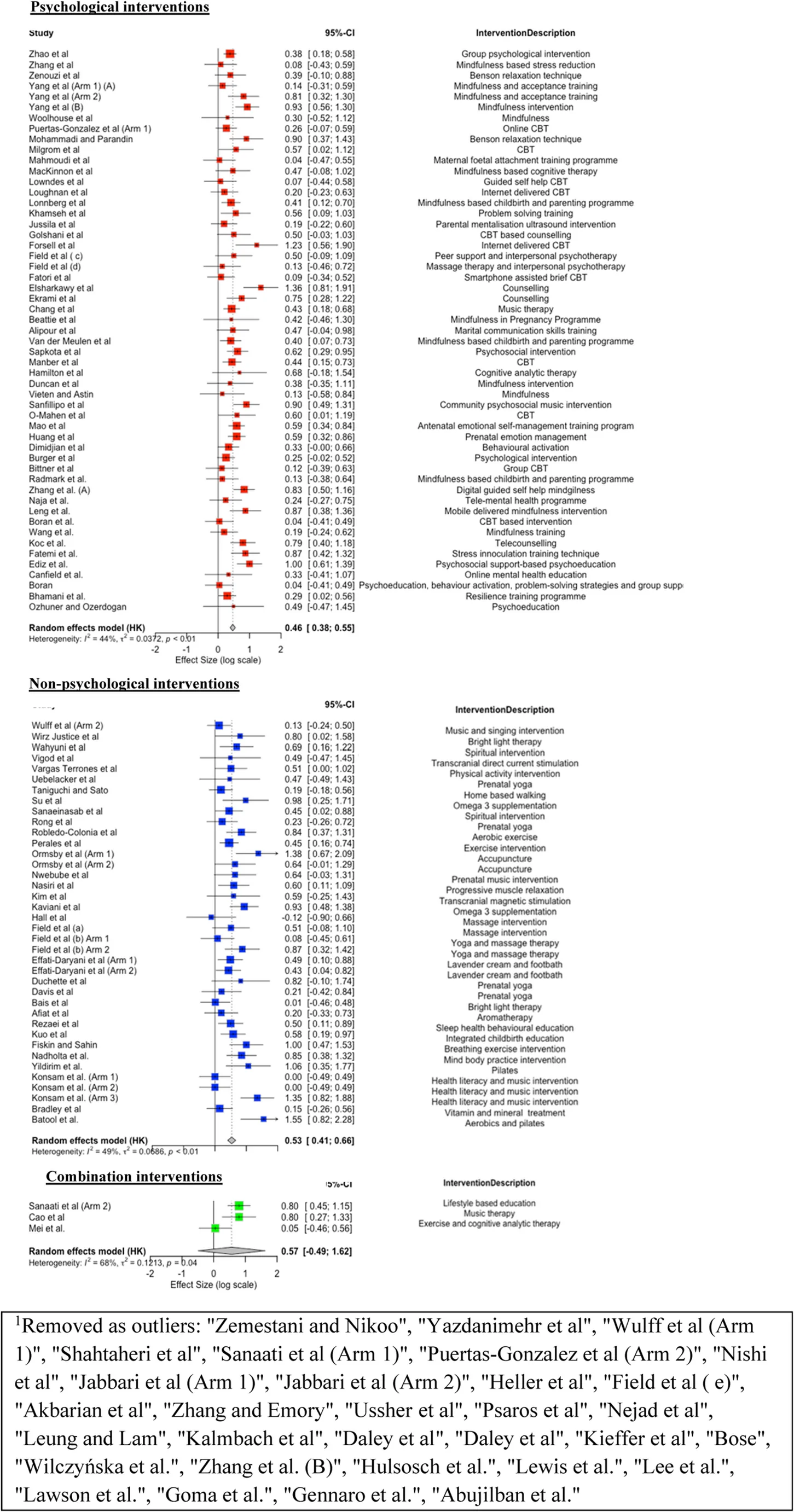
***Forest plot displaying effect sizes of depression scores post-intervention compared with control (outliers removed)***,*** with 95% intervals***,*** and overall pooled effect size and estimates of heterogeneity***

We undertook additional subgroup analyses on the studies presented in Fig. 2. No significant differences were observed between the three groups, that is, psychological, non-psychological and combination treatments (*p* = 0.63). Further subgroup analyses were undertaken to assess numerical differences in efficacy based on intervention type.

Both psychological and non-psychological interventions showed moderate efficacy (SMD = 0.46, 95% CI 0.4; 0.55 *p* < 0.005 and SMD 0.53, 95% CI 0.41; 0.65, *p* < 0.0005). Finally, interventions that utilized a combination of the two also showed comparable efficacy (SMD 0.57 95% CI 0.08; 1.05, *p* < 0.05).

Another subgroup analysis was undertaken to identify whether differences in efficacy arose as a result of intervention format, measure-; these differences were not significant (*p* = 0.88). As above, all intervention formats had similar numerical efficacy.

85 arms utilised a self-report measure to assess depressive symptomology, and 9 arms utilised a clinician rated outcome measure. The difference was not significant (*p* = 0.64). although numerically the efficacy of interventions was higher for clinician-reported outcomes (SMD 0.58; 95% CI 0.31; 0.85), as compared to self-reported outcomes (SMD 0.48; 95% CI 0.42; 0.56). However, given that only 9 studies reported on clinician-rated outcome, these results need to be interpreted with caution. All effect sizes were comparable, and results of subgroup analyses are summarized in Table 1.

**Table 1:** Tabular visualisation of subgroup analyses (when outliers have been removed)

	K	SMD	*P* value	95% CI	*P* value (subgroup differences)
Intervention type					
Psychological	53	0.46	*p* < 0.0001	0.4; 0.55	0.63
Non psychological	38	0.53	*p* < 0.0001	0.41; 0.65	
Combination	3	0.57	*p* < 0.05	0.08, 1.05	
**Intervention format**					
In person	62	0.5	*p* < 0.0001		0.9
Online/digital	21	0.51	*p* < 0.0001		
Monitored self-administration	9	0.41	*p* < 0.05		
Combination	2	0.56	*p* < 0.05	0.15 0.97	
**Measure type**					
Clinician	9	0.58	*p* < 0.0001	0.31; 0.85	0.5
Self-report	85	0.48	*p* < 0.0001	0.42; 0.56	
**Income region**					
High income	44	0.36	*p* < 0.0001	0.3; 0.43	**0.0007**
Upper-middle income	40	0.55	*p* < 0.0001	0.45; 0.64	
Lower-middle income	9	0.7	*p* < 0.05	0.34; 1.09	
Low income	1	0.9	*p* < 0.0001	0.5; 1.31	
**Geographical region**					
East Asia and Pacific	26	0.47	*p* < 0.0001	0.34; 0.6	0.16
Europe and Central Asia	22	0.43	*p* < 0.0001	0.29; 0.58	
Latin America and Caribbean	2	0.46	*p* < 0.22	−0.27; 1.19	
Middle East and North Africa	18	0.58	*p* < 0.0001	0.45; 0.72	
North America	17	0.41	*p* < 0.0001	0.32; 0.51	
South Asia	8	0.63	*p* < 0.05	0.25; 1.02	
Sub Saharan Africa	1	0.9	*p* < 0.0001	0.49; 1.31	
**Control Group Type**					
Active control	16	0.42	*p* < 0.0001	0.26; 0.59	0.59
Placebo	7	0.5	*p* < 0.001	0.22; 0.76	
Sham control	2	0.55	*p* < 0.0001	0.45; 0.64	
Unspecified	2	0.94	*p* < 0.05	0.11; 1.7	
Routine antenatal care	67	0.5	*p* < 0.0001	0.42 0.57	

### Biomarker analysis

**Fig. 3:**
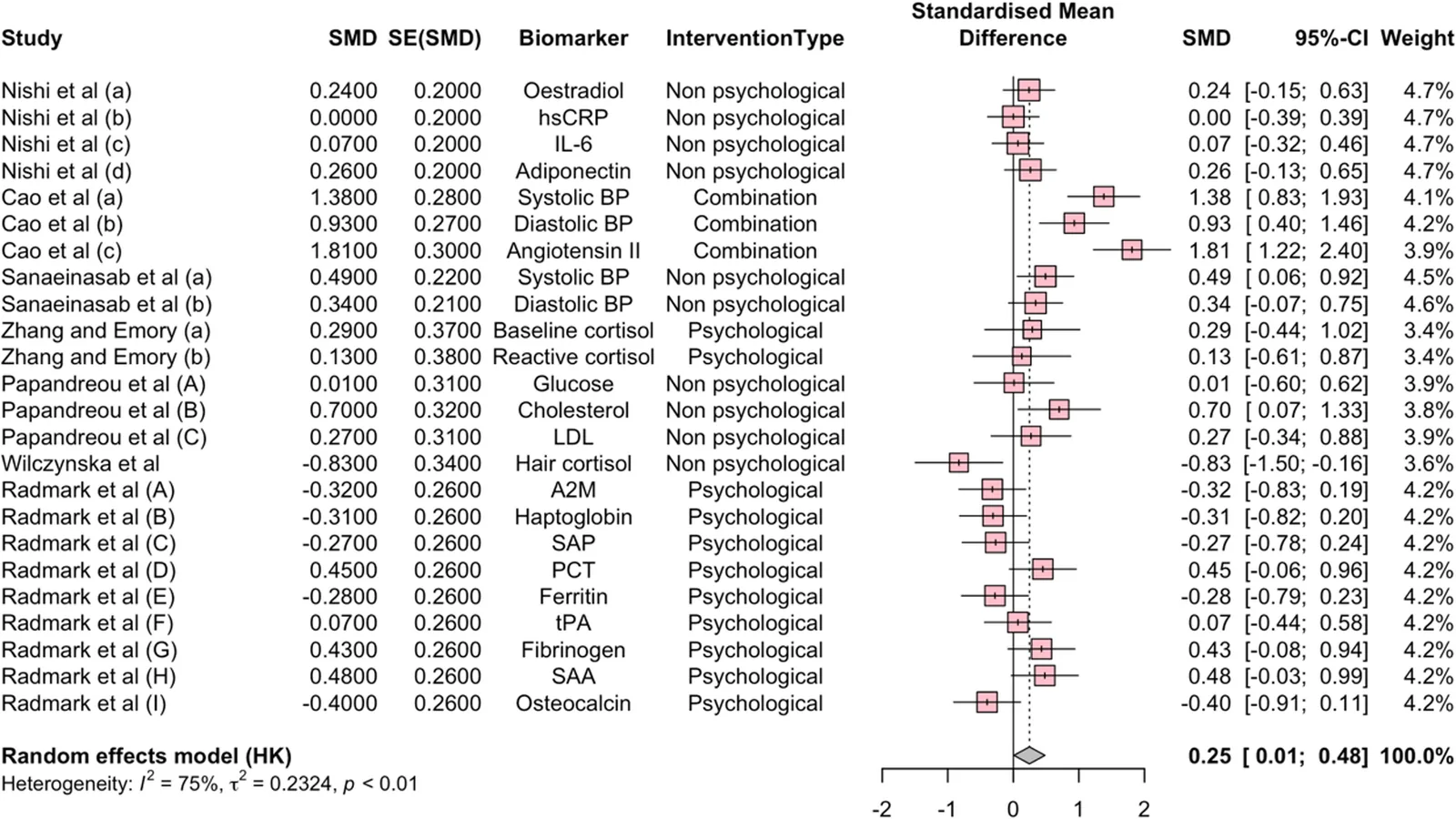
Forest plot displaying effect sizes of biomarker levels post-intervention compared with control, with 95% intervals, and overall pooled effect size and estimates of heterogeneity

Within the larger dataset, a small number of studies reported on biological outcomes. Effect sizes were pooled to undertake a meta-analysis to determine the ability of interventions to differentially affect the post-intervention levels of the various biomarkers, i.e., we tested for post-intervention differences between the intervention and the control group. Biomarkers analysed included oestradiol, high-sensitivity C-reactive protein (hsCRP), interleukin (IL)−6, adiponectin, cortisol, and blood pressure. Overall, 7 studies were included, and we acknowledge the physiological heterogeneity of pooled biomarkers in our analysis. Results of the meta-analysis are displayed in Fig. 3.

For the purpose of simplicity, positive effect sizes indicate that the differences in the post-intervention levels of the biomarker favoured the intervention, i.e., suggested lower stress levels in the intervention group (see Supplementary Table 3).

Together, the interventions affected the biomarkers in directions consistent with a “stress reduction” effect, suggesting that biological changes accompany the mental health improvement (SMD = 0.25, 95% CI 0.01; 0.48). Music therapy had the largest effect size (Cao et al. 2016) on systolic (SMD 1.38, 95% CI 0.83; 1.93) and diastolic blood pressure (SMD 0.93, 95% CI 0.4; 1.46, as well as angiotensin II (SMD 1.81, 95% CI 1.22; 2.4). Some studies however did not show such an effect, for example, in the study by (Wilczynska et al. 2023), the levels of cortisol were higher in the intervention group than in the control group.

As these biomarkers were varied, as expected, levels of heterogeneity were substantially high (I^2^ = 73%). However, we undertook another meta-analysis after accounting for 3 outliers (Cao et al. (a), Cao et al. (b), and Wilczynska et al.), and the overall results remain unchanged (SMD = 0.17, 95% CI 0.01; 0.33). Results of this analysis are summarised in Supplementary Material Fig. 2.

### Publication bias

Publication bias was assessed by visually inspecting funnel plots, administering Egger’s test to identify plot asymmetry, and using Duval and Tweedie’s trim and fill method (Duval and Tweedie 2000) to assess bias. In the main analysis including all studies, publication bias was identified using all three methods. When outliers were removed, no bias was detected. Results of these tests are displayed in the Supplementary Material Figs. 3.

**Fig. 4:**
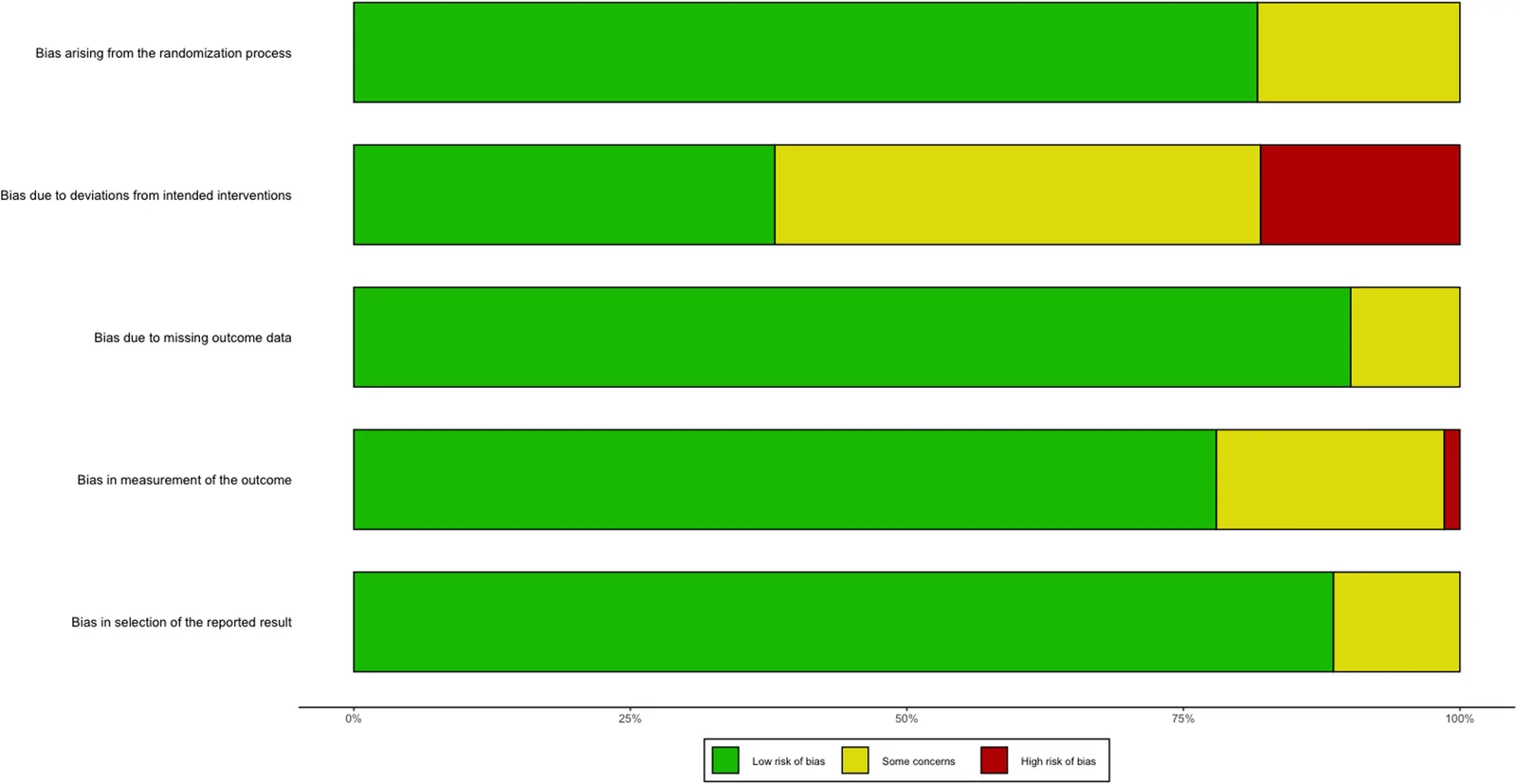
Summary plot of Risk of Bias Domains assessed using the RoB 2 tool, including the following domains: (**a**) Bias arising from the randomization process, (**b**) Bias due to deviations from intended interventions, (**c**) Bias due to missing outcome data, (**d**) Bias in measurement of the outcome, and (**e**) Bias in selection of the reported result

As per the RoB V2 tool, the following domains of publication bias were assessed: (a) Bias arising from the randomization process, (b) Bias due to deviations from intended interventions, (c) Bias due to missing outcome data, (d) Bias in measurement of the outcome, (e) Bias in selection of the reported results. Figure 4 summarizes the level of risk of bias for each domain. Overall, studies had a low risk of bias, however the domain with the highest risk was domain (b).

## Discussion

To our knowledge, this is the first meta-analysis synthesising the entire evidence base of interventions in the treatment of AD, along with a focus on biomarkers influenced by the interventions. Results of our analysis found an overall moderate treatment effect (SMD = 0.65, *p* < 0.0001) of interventions when compared to control conditions over depressive symptomology. Given the substantial levels of heterogeneity (I^2^ = 88%), our secondary analysis, eliminating outliers, still found a clear treatment effect (SMD = 0.5, *p* < 0.0001), with lower levels of heterogeneity (I^2^ = 46.3%).

Intervention types were varied, including, but not limited to, psychotherapy, yoga, CBT, music therapy, mindfulness, Omega-3 supplementation, and bright light therapy. Interestingly, we found no differences in efficacy between psychological and non-psychological interventions. Previous meta-analyses across specific intervention types have shown differences in effect sizes, for example 0.41 for exercise (Zhang et al. 2024), and 0.76 for mindfulness (Li et al. 2022)), although these were calculated in different meta-analyses. Contrary to these findings, in our common meta-analysis we did see that psychological, non-psychological and combination interventions had comparable efficacy (SMD = 0.46, 0.53, and 0.57 respectively). While part of this can be explained by the common methodology for the estimation across all types of studies, we also postulate that this might be in part due to the range of risk factors in the development of antenatal depression, as seen from the HappyMums clinical study (Priestley et al. 2026), which include previous mental health history, lack of social support, and history of fertility issues. Different intervention types, like peer support, CBT, and mindfulness might address these risk factors during treatment, which might explain similar improvements across intervention types. In terms of intervention format, digitally delivered interventions, such as iCBT, use the same principles as interventions delivered in person, and therefore have the same therapeutic mechanisms at play.

Our review indicates the potential of non-psychological interventions, such as self-help, or Omega 3 supplementation, to be used as adjunctive therapies, for added therapeutic value, or as effective low-intensity interventions while waiting to access psychological support. Indeed, non-psychological interventions might be offered as “first line” for mild depressive symptoms, to offer treatment while reducing burden on healthcare systems, and reserving higher intensity or specialized psychological intervention for moderate to severe depressive symptomology. This is in line with current National Institute for Health and Care Excellence (NICE) guidelines in the UK, where self-help is offered for subthreshold depressive symptoms, or mild to moderate depression, while high intensity psychological intervention is provided to women with moderate or severe depression in pregnancy (*Antenatal and Postnatal Mental Health: Clinical Management and Service Guidance*, 2020).

We acknowledge that the range of interventions is very wide, ranging from psychotherapy, to aromatherapy, and bright light therapy, with different therapeutic mechanisms at play. Therefore, we explored through subgroup analyses whether efficacy across intervention types differed. Indeed, as per the analysis on intervention type, we did not find significant subgroup differences, as shown in Table 1. Furthermore, we agree with similar work in the field (Dennis and Allen 2008; Wu et al. 2025) on the limited interpretability of specific intervention types like aromatherapy for antenatal depression, due to limited evidence. Therefore, we do feel that the pooled effect sizes need to be interpreted with caution, due to the heterogeneity levels in our analysis. Nevertheless, we believe that the rich set of subgroup analyses across intervention type and format provide a thorough perspective on the evidence base.

Moreover, the four studies that integrated both psychological and non-psychological aspects also showed comparable efficacy. One study combined exercise with CBT (Mei et al. 2024). Given that both CBT (Pettman et al. 2023) and physical activity (He et al. 2023) show moderate to high efficacy in managing AD, combining the two seems to be a promising approach for treating depression in pregnancy. However, it is important to consider clinician guidance for pregnant people before undertaking different forms of physical activity. In the absence of clear superiority of one approach over the other, patient choice and lifestyle and medical considerations should guide the treatment choice, while future research should investigate predictors that could personalise the best treatment for the individual patient. As pregnancy is such a biologically sensitive period, identifying, for example, biomarkers associated with a response to interventions, could provide a clinically relevant personalised approach to the choice of treatment, as is happening with inflammatory biomarkers in the context of depression outside pregnancy (Miller et al. 2025).

In terms of intervention format, we found some suggestive evidence that interventions delivered digitally/online were as effective as interventions delivered in person. These findings are in line with previous meta-analyses undertaken to review the efficacy of digital interventions in treating perinatal depression in general and postnatal depression in particular (Ansaari et al. 2024; Ashford et al. 2016; Li et al. 2023). One reason for this could be that digital interventions might be easier to access for pregnant (or newly postnatal) women, who might experience physical barriers to accessing interventions delivered in person and instead can attend sessions online. For example, we have recently shown that an online singing intervention is effective in reducing depression and anxiety in postnatal depression (Bind et al. 2023).

As for combined interventions, combined delivery formats also showed comparable efficacy to those employing one intervention format. Integrating the two provides high levels of flexibility for participants, with digital options available in between in person sessions, with more frequent support from intervention providers. We propose that having a flexible approach also allows for facilitators to engage with participants in-between physically delivered sessions and therefore might be associated with higher levels of participant engagement.

It is important to note that our systematic review of the literature did not yield results of randomised controlled trials assessing the impact of pharmacological interventions on AD which met our inclusion criteria. This is because, to date, no RCT has been conducted to prospectively assess the efficacy of antidepressant medication to treat depressed pregnant women and their subsequent impact (Howland 2013). This was already discussed more than 10 years ago by Howland (2013), who said that research undertaken globally interprets and emphasises data somewhat differently, and does not provide consistent guidance either for patients or clinicians. Indeed, there have been no changes since, partly due to the ethical and practical reasons associated with the potentially increased risk of trialing pharmacotherapy during the perinatal period (Andrade 2017). RCTs have been conducted assessing medications like fluoxetine (Milgrom et al. 2015)and paroxetine (Milis, et al. 2004) to treat postnatal depression, but at the time of data extraction, no RCT had assessed pharmacotherapy for antenatal depression.

Our analysis found that most studies used self-reported outcome measures. Previous literature on the efficacy of psychotherapy on depression has shown that self-report measures are associated with lower effect sizes when compared with clinician rated outcomes (Cuijpers et al. 2010). Interestingly, our results were in the same direction, albeit the difference was not statistically significant. The number of studies with clinician rated measures (k = 9) is smaller than those with self-report measures (k = 85), leading to a numerically larger effect size (SMD = 0.58 for clinician rated measures and 0.48 for self-report measures). Therefore, further research into using both clinician- as well as self-report measures is warranted to truly understand intervention efficacy. It is important to emphasize, however, that clinician rated measures are indeed more objective and less susceptible to bias, especially when the clinician or outcome assessor is blinded to intervention allocation, and this might improve the precision of the measurement and thus the effect size.

Comparisons based on geographical regions did reveal significant differences, with studies conducted in the Middle East, North Africa, and Sub-Saharan Africa having the highest effect sizes. Given that these regions fall under the Upper Middle to the Lower Middle-income countries (LMIC), this indicates that the interventions were most effective at managing AD symptomology despite financial and economic resource constraints. In fact, subgroup analyses of efficacy by income region also revealed subgroup differences. One reason for this difference could be the difference in the standard of care between High-income countries (HIC) and LMICs. Primary care and mental health services in LMICs are in general underfunded, and therefore, those needing access to care might not be treated. Therefore, when taking part in a trial aimed to manage depressive symptomology, intervention can lead to significant improvements, if compared with a very limited or non-existent “control” care as usual. In fact, as also highlighted by Cuijpers et al. (2018), care as usual in Western or High-income countries might already include access to community mental health services or other routine treatment pathways, while this might not be the case in LMICs, where usual care as usual means no access to mental health care. Therefore, it is indeed possible that the intervention group show larger effect sizes because they receive treatment, while the care as usual group does not have any access. Given that the prevalence of antenatal depression is highest in LMICS (Roddy Mitchell et al. 2023), it is important to emphasise that any form of intervention could yield larger improvements in depressive symptomology in these women.

In our analyses, high levels of heterogeneity were expected due to the wide range of intervention types, format and outcomes, as well as delivery countries and cultures. This has been seen in previous meta-analyses of interventions in the context of antenatal depression, which also indeed reported moderate to high levels of heterogeneity (Liu et al. 2023; Yasuma et al. 2020) citing similar reasons to ours, such as differences in outcome measures (Yasuma et al. 2020), intervention format, and duration (Liu et al. 2023). Societal factors also may have contributed significantly to the levels of heterogeneity here, as we did see significant subgroup differences based on income regions. Furthermore, our synthesis of the literature found numerous types of outcome measures being used, such as the EPDS, BDI, PHQ-9, CES-D, and clinician rated measures. These outcome measures, though validated for use during the perinatal period (Sambrook Smith et al. 2022), can lead to between study heterogeneity, due to aspects such as cut off scores, method of administration, and constructs measured. Differences in control conditions might be an additional factor which contributes to between-study heterogeneity. Certain studies utilized an active or placebo control, while others had participants on a waitlist or treatment as usual control. Therefore, this variation might have contributed to study heterogeneity. Moreover, “treatment as usual” might differ worldwide: while for some, this means access to community or specialist mental health teams, for others, it means no treatment at all. That said, we did not find significant subgroup differences based on control group type. For baseline severity, studies, such as required participants to have a diagnosis of depression, while for others, there was no baseline severity as inclusion. Therefore, while some studies have a sample who met criteria for current antenatal depression, others included low mood, without having to meet threshold to be included in the study. While examining baseline depression scores, some studies, such as Chang M.-Y. et al. (2008) had a mean baseline EPDS score of approximately 12, while others, like Fontein-Kuipers et al. (2016) had a mean baseline EPDS score of around 4.5, highlighting that participants did not meet criteria for depression. Reassuringly, our risk of bias assessment found low risk for. “Bias arising from the randomisation process”.

Out of 115 RCTs, 7 reported on biological outcomes. Pregnancy is a time of numerous biological changes, and it is important to note that between-group effect sizes post-interventions are confounded by normal biomarker changes across this period (Radmark et al. 2023), despite attempts to mitigate this by focusing on RCTs. Second, biomarkers are examined at a single time point post-intervention and may be influenced by gestational timing or normal pregnancy-related changes. Given the varied range of reported biomarkers, levels of between-study heterogeneity were expectedly high. Despite this, our results highlight the overarching biopsychosocial impact that interventions have on antenatal depression, affecting both depression and biological mechanisms linked to stress, and usually in the same direction, i.e., with a significant overall “stress-reduction” effect. Overall, the biological evidence is heterogeneous and does not point to a single mechanistic pathway. HPA-axis and immune dysfunction are widely implicated in antenatal depression (Osborne et al. 2018), suggesting that stress-regulation mechanisms may represent one important intervention target. Some studies showed changes in stress-related biomarkers such as cortisol and blood pressure (Cao et al. 2016; Sanaeinasab et al. 2021) while others indicated influences on reproductive endocrine factors (e.g., oestradiol) (Nishi et al. 2020) or cardiometabolic markers (lipids and glucose) (Papandreou et al. 2023), which may interact with stress-physiology pathways. Although few intervention-specific differences were observed in immune markers, inflammation is still thought to contribute to perinatal depression, highlighting the need for more intervention studies that consistently measure biological biomarkers to clarify underlying mechanisms. We would like to reiterate that pooling physiologically different biomarkers has indeed led to substantial levels of heterogeneity, while at the same time offering the opportunity to explore a unifying conceptual framework based on, broadly speaking, the effects of intervention on stress-related mechanism. While, based on this meta-analysis, we cannot point to a single mechanistic pathway, we do add to the growing body of evidence that HPA axis and immune system dysregulation are implicated in antenatal depression, and offer some promising signal for the role of cardiovascular indicators such as blood pressure and Angiotensin II. Thus, interventions targeting stress reduction might provide a mechanism to manage depressive symptomology in pregnancy, but this interpretation is limited by the largely heterogeneous nature of included studies.

Similar work on this topic was done by Kwok and colleagues in 2025 (Kwok et al. 2025), wherein they reviewed major biomarkers involved in MDD, which also had associations with perinatal depression in the context of treatment response. We now build on this, drawing data from published work, and identifying specific biomarkers that change as a result of treatment. This is with the caveat that we did not do this from the perspective of examining biomarkers predicting response, but rather seeing the impact that interventions had on biomarkers. Examining intervention efficacy from a biological and a psychological lens can help clinicians with providing a more personalized, precision-based treatment approach in antenatal depression.

One study with the highest effect sizes (Cao et al. 2016) delivered a music therapy intervention, and identified substantial reduction in blood pressure levels and in angiotensin II, a biomarker which has emerged as a critical hormone affecting the function of key organs like the kidney, heart, and the brain (Patel and Mehta 2012). This finding highlights the efficacy of arts in health interventions in treating antenatal depression and their associated biomarkers. Seeing as previous music and singing based interventions have shown promising results in the treatment of postnatal depression (Bind et al. 2025; Fancourt and Perkins 2018), further research into this intervention type for antenatal depression could prove to be a promising pathway from pregnancy to the first year postpartum.

Once again, along the same trend as the clinical outcomes analysis, we saw significant levels of heterogeneity in the biomarker effects (I^2^ = 75%), which reduced (I^2^ = 45%) once we removed outliers. Of course, our search uncovered different types of biomarkers, from cholesterol to blood pressure, hsCRP, and Angiotensin II, all of which have distinct features, changing during pregnancy. Indeed, there might have been numerous ways of extracting samples (blood versus saliva), along with different mechanisms being tested, which contribute significantly to heterogeneity. Our analysis was exploratory in nature, and future analyses should examine specific biomarker categories and their association with treatment outcomes.

In our primary analysis with all studies included, we identified publication bias through three different methods, along with substantial levels of between-study heterogeneity. Indeed, as we expected, upon removing outliers, no publication bias was found, and this also led to a reduction in levels of heterogeneity. Nevertheless, it is possible that studies with very high effect sizes were likely to be influenced by publication bias and their results must be interpreted with caution.

The results of our analysis need to be interpreted in the context of certain limitations, the most important being the substantial levels of heterogeneity in the primary analysis with 115 trials, which could have had significant impact on effect sizes. Moreover, in the context of search methodology, though we conducted a robust search across different databases, we did not extract papers from the Cochrane Library and Web of Science. This is because our data extraction took place on OVID, which allows for searching across key databases relevant to perinatal mental health, such as Embase, PsycINFO, Medline, and the Maternity and Infant Care Database (MIDIRS). Reassuringly, when cross checking against other meta- analyses on antenatal depression, we covered all of their included studies. Nevertheless, we cannot absolutely exclude the possibility that using additional databases might have led to more studies identified. While the review was not registered at its inception in 2022, we followed the declared objectives of HappyMums as described in the funded application (Task 3.5) and the protocol paper (Biaggi et al. 2025). Specifically, the protocol paper states that “a literature-based meta-analysis investigating factors associated with response to interventions for depression in pregnancy will also be conducted”, and that “sociodemographic (…), environmental (…) and biological factors (e.g., … inflammation, metabolism-related biomarkers/metabolites, and neuroendocrine-related biomarkers) will be analysed”. The analytic decisions were guided by these overarching objectives as well as the need to dissect the effects of treatment features (e.g., intervention type and format) and socio-demographic/environmental conditions (e.g., income and geographical regions). Removal of grey literature was intentional, to ensure we had sufficient information as normally reported in published research in order to be consistent across risk of bias assessments. We do understand that exclusion of grey literature from meta analyses can have implications on exaggerated effect sizes (McAuley et al. 2000) however given the wide range of studies currently included in our analysis, and the high resulting variability, we believe this provides an adequate synthesis of available published literature. Given that unpublished studies in specific populations represent a small proportion of included studies in reviews (Hartling et al. 2017), we felt the above rationale appropriate to exclude grey literature.

In conclusion, this first-of-a-kind meta-analysis demonstrates the efficacy of varied interventions in the management of depression during pregnancy. Though results seem promising, they must be interpreted with caution owing to the substantial levels of heterogeneity. Future research will need to clarify whether combining the most effective interventions can lead to even stronger improvement, or if adding biological data to the clinical assessment could facilitate personalized, more effective interventions.

## Supplementary Information

Below is the link to the electronic supplementary material.

Supplementary Material 1 (DOCX 40.3 KB)

Supplementary Material 2 (DOCX 21.7 KB)

Supplementary Material 3 (DOCX 23.5 KB)

Supplementary Material 4 (DOCX 18.2 KB)

Supplementary Material 5 (DOCX 218 KB)

Supplementary Material 6 (DOCX 364 KB)

Supplementary Material 7 (DOCX 1.03 MB)

## Data Availability

Data is provided within the manuscript or supplementary information files.

## Declarations

### Clinical trial number

Not aplicable.
