# Supplementary material for: The evidence base of interventions to treat antenatal depression: a meta-analysis of randomized controlled trials
Source: Arch Womens Ment Health. 2026 Jul 3;29(4):103. doi: 10.1007/s00737-026-01723-0 (PMC13331926; doi:10.1007/s00737-026-01723-0)
Supplement: Supplementary file 1 — Supplementary Material 1 (DOCX 40.3 KB) [file 737_2026_1723_MOESM1_ESM.docx]

| **Author, Year, Country** | **Intervention Details (N)** | **Control Group (N)** | **Measure Used** | **Effect size (SMD, 95% CI)** | **Risk of Bias** |
| --- | --- | --- | --- | --- | --- |
| Afiat et al, 2022, Iran | Rose aromatherapy and metoclopramide for nausea (20) | Placebo control (20) | HADS | 0.2 [-0.33; 0.73] | Low |
| Alipour et al, 2020, Iran | Marital communication skills training (27) | Active control: 2 sessions of childbirth preparation course (27) | GHQ | 0.47 [-0.04; 0.98] | Low |
| Bais et al, 2020, Netherlands | Bright light therapy (33) | Dim red light therapy (34) | HAM-D | 0.01 [-0.46; 0.48] | Low |
| Batool et al, 2023, Pakistan | Aerobic exercise intervention (19) | Pilates exercise group (19) | CES-D | 1.55 [0.82; 2.28] | Some concerns |
| Beattie et al, 2017, Australia | Mindfulness based intervention (9) | Pregnancy support programme (11) | EPDS | 0.42 [-0.46; 1.3] | Some concerns |
| Bhamani et al, 2024, Pakistan | Accessible resilience training intervention (100) | Routine prenatal care (100) | EPDS | 0.29 [0.02; 0.56] | Some concerns |
| Bittner et al, 2014, Germany | CBT (21) | Routine prenatal care (53) | EPDS | 0.12 [-0.39; 0.63] | Some concerns |
| Boran et al, 2023, Turkey | CBT (35) | Routine prenatal care (38) | EPDS | 0.04 [-0.41; 0.49] | Some concerns |
| Bradley et al, 2024, New Zealand | Micronutrient supplementation (44) | Placebo control (44) | MADRS | 0.15 [-0.26; 0.56] | Low |
| Burger et al, 2019, the Netherlands | CBT (97) | Routine prenatal care (104) | EPDS | 0.25 [-0.02; 0.52] | Low |
| Canfield et al, 2023, USA | Online mental health intervention(14) | Enhanced routine prenantal care(14) | EPDS | 0.33 [-0.41; 1.07] | Some concerns |
| Cao et al, 2016, China | Music therapy (30) | Routine prenatal care (30) | HAM-D | 0.8 [0.27; 1.33] | Some concerns |
| Chang et al, 2007, Taiwan | Music therapy (116) | Routine prenatal care (120) | EPDS | 0.43 [0.18; 0.68] | Low |
| Davis et al, 2015, USA | Prenatal yoga (19) | Routine prenatal care (20) | EPDS | 0.21 [-0.42; 0.84] | Some concerns |
| Dimidjian et al, 2017 | Behavioural activation (70) | Routine prenatal care (68) | PHQ-9 | 0.33 [-0.00; 0.66] | Low |
| Duchette et al, 2021, USA | Prenatal yoga (10) | Routine prenatal care (9) | POMS | 0.82 [-0.1; 1.74] | Some concerns |
| Duncan et al, 2017, USA | Mindfulness based childbirth preparation course (15) | Standard childbirth education course (14) | CES-D | 0.38 [-0.35; 1.11] | Some concerns |
| Ediz et al, 2024, Turkey | Psychoeducation (50) | Routine prenatal care (55) | EPDS | 1 [0.61; 1.39] | High |
| Effati Daryani et al, 2015, Iran (Arm 1) | Lavender cream with footbath (47) | Placebo cream (47) | DASS | 0.49 [0.10; 0.88] | Low |
| Effati Daryani et al, 2015, Iran (Arm 2) | Lavender cream (47) | Placebo cream (47) | DASS | 0.43 [0.04; 0.82] | Low |
| Ekrami et al, 2019, Iran | Counselling (37) | Unspecified control (39) | EPDS | 0.75 [0.28; 1.22] | Some concerns |
| Elsharkawy e t al, 2021, Egypt | Counselling (3) | Unspecified control (30) | DASS | 1.36 [0.81; 1.91] | Some concerns |
| Fatemi et al, 2023, Iran | Virtual stress inoculation training techniques (40) | Semi attendance stress inoculation training (40) | PSS | 0.87 [0.42; 1.32] | Low |
| Fatori et al, 2022, Brazil | Online CBT (37) | Phone app (44) | EPDS | 0.09 [-0.34; 0.52] | Some concerns |
| Field et al (a), 2007, USA | Partner delivered massage (23) | Unspecified control (24) | CES-D | 0.51 [-0.08; 1.10] | High |
| Field et al (b) Arm 1, 2011, USA | Prenatal yoga (28) | Routine prenatal care (28) | CES-D | 0.08 [-0.45; 0.61] | High |
| Field et al (b) Arm 2, 2011, USA | Massage therapy (28) | Routine prenatal care (28) | CES-D | 0.87 [0.32; 1.42] | High |
| Field et al (c), 2013, USA | Interpersonal psychotherapy (22) | Support group (22) | CES-D | 0.5 [-0.09; 1.09] | High |
| Field et al (d), 2008, USA | Group interpersonal psychotherapy (21) | Group interpersonal psychotherapy with massage (22) | CES-D | 0.13 [-.0.46; 0.72] | High |
| Fiskin and Sahin, 2018, Turkey | Diaphragmatic breathing (30) | Routine prenatal care (30) | DASS | 1 [0.47; 1.53] | Some concerns |
| Forsell et al, 2017, Sweden | Internet based CBT (22) | Routine prenatal care (20) | MADRS | 1.23 [0.56; 1.9] | Some concerns |
| Golshani et al, 2021, Iran | CBT based counselling (28) | Routine prenatal care (28) | EPDS | 0.5 [-0.03; 1.03] | Some concerns |
| Hall et al, 2020, Australia | Partner delivered massage (13) | Self directed stretching (11) | DASS | -0.12 [-0.9; 0.66] | High |
| Hamilton et al, 2020, UK | Cognitive analytic therapy (20) | Routine prenatal care (19) | EPDS | 0.68 [-0.18; 1.54] | High |
| Huang et al, 2015, China | Antenatal emotion management programme (100) | Routine prenatal care (100) | PHQ-9 | 0.59 (0.32; 0.86] | Some concerns |
| Jussila et al, 2020, Finland | Parenatal mentalization intervention (46) | Routine prenatal care (44) | EPDS | 0.19 [-0.22; 0.6] | Some concerns |
| Kaviani et al, 2014, Iran | Omega 3 supplementation (40) | Placebo olive oil pill (40) | BDI | 0.93 [0.48; 1.38] | Low |
| Khamseh et al, 2019, Iran | Problem solving skills training (35) | Routine prenatal care (35) | BDI | 0.56 [0.09; 1.03] | Some concerns |
| Kim et al, 2019, USA | Transcranial magnetic stimulation (11) | Sham stimulation (11) | HAM-D | 0.59 [-0.25; 1.43] | Low |
| Koc et al, 2022, Turkey | Telephone counselling (50) | Routine prenatal care (50) | HADS | 0.79 [0.4; 1.18] | Some concerns |
| Konsam et al, 2023, India (Arm 1) | Relaxing music intervention (32) | Routine prenatal care (32) | EPDS | 0.00 [-0.49; 0.49] | Low |
| Konsam et al, 2023, India (Arm 2) | Comprehensive health literacy intervention (32) | Routine prenatal care (32) | EPDS | 0.00 [-0.49; 0.49] | Low |
| Konsam et al, 2023, India (Arm 3) | Combination of music and health literacy (32) | Routine prenatal care (32) | EPDS | 1.35 [0.82; 1.88] | Low |
| Kuo et al, 2022, Taiwan | 8 week childbirth education programme (53) | Routine prenatal care (53) | EPDS | 0.58 [0.19; 0.97] | Some concerns |
| Leng et al, 2023, China | Mobile delivered mindfulness intervention programme (35) | Web based perinatal education programme (35) | EPDS | 0.87 [0.38; 1.36] | Low |
| Lonnberg et al, 2019, Sweden | Mindfulness Based Childbirth and Parenting Programme (75) | Lamaze (89) | EPDS | 0.41 [0.12; 0.7] | High |
| Loughnan et al, 2018, Australia | Internet delivered CBT (36) | Usual care (41) | EPDS | 0.20 [-0.23; 0.63] | Some concerns |
| Lowndes et al, 2018, Australia | CBT (30) | Waitlist control (30) | EPDS | 0.07 [-0.44; 0.58] | High |
| Mahmoudi et al, 2021, Iran | Maternal foetal attachment intervention (29) | Routine prenatal care (32) | EPDS | 0.04 [-0.47; 0.55] | Some concerns |
| MaKinnon et al, 2021, USA/ Canada | Mindfulness Based Cognitive Therapy (28) | Routine prenatal care (32) | EPDS | 0.47 [-0.08; 1.02] | High |
| Manber et al, 2019, USA | CBT for insomnia(89) | Modified pseudodesensitization therapy for insomnia (90) | EPDS | 0.44 [0.15; 0.73] | Some concerns |
| Mao et al, 2012, China | Emotional self management group training (120) | Routine prenatal care (120) | PHQ-9 | 0.59 [0.34; 0.84] | Low |
| Mei et al, 2023, China | Exercise and cognitive analytic therapy (30) | Routine prenatal care (30) | HAM-D | 0.05 [-0.46; 0.56] | Some concerns |
| Milgrom et al, 2015, Australia | CBT (27) | Usual care (27) | BDI | 0.57 [0.02; 1.12] | Some concerns |
| Mohammadi and Parandin, 2019, Iran | Benson’s Relaxation technique (30) | Routine prenatal care (30) | DASS | 0.9 [0.37; 1.43] | High |
| Nadholta et al, 2023, India | Yoga (34) | Usual care (43) | DASS | 0.85 [0.38; 1.32] | Some concerns |
| Naja et al, 2022, Qatar | Telemental health psychosocial intervention (29) | Routine prenatal care (29) | EPDS | 0.24 [-0.27; 0.75] | Low |
| Nasiri et al, 2018, Iran | Progressive muscle relaxation (33) | Routine prenatal care (33) | DASS | 0.6 [0.11; 1.09] | Some concerns |
| Nwebube et al, 2017, UK | Music based intervention (20) | Quiet sitting (16) | EPDS | 0.64 [-0.03;1.31] | Some concerns |
| Ormsby et al, 2020, Australia (Arm 1) | Accupuncture (19) | Routine prenatal care (19) | EPDS | 1.38 [0.67; 2.09] | High |
| Ormsby et al, 2020, Australia (Arm 2) | Progressive muscle relaxation (19) | Routine prenatal care (19) | EPDS | 0.64 [-0.01; 1.290 | High |
| Ozhuner and Ozerdogan, 2024, Turkey | Psychoeducation (45) | Routine prenatal care (46) | EPDS | 0.49 [-0.47; 1.45] | Some concerns |
| O’Mahen et al, 2013, USA | Cognitive Behavioural therapy (21) | Routine prenatal care (23) | BDI | 0.6 [0.01; 1.19] | Low |
| Perales et al, 2015, Spain | Exercise based intervention (90) | Routine prenatal care (77) | CES-D | 0.45 [0.16; 0.74] | Some concerns |
| Puertas-Gonzalez et al, 2022, Spain (Arm 1) | Online CBT group intervention (70) | Routine prenatal care (68) | SCL-90-4 | 0.26 [-0.07; 0.59] | Some concerns |
| Radmark et al, 2023, Sweden | Mindfulness based childbirth and parenting programme (26) | Lamaze (34) | EPDS | 0.13 [-0.38; 0.64] | Some concerns |
| Rezaei et al, 2014, Iran | Sleep health behaviour education (48) | Routine prenatal care (48) | BDI | 0.5 [0.11; 0.89] | Low |
| Robledo Colonia et al, 2012, Colombia | Exercise based intervention (37) | Routine prenatal care (37) | CES-D | 0.84 [0.37; 1.31] | High |
| Rong et al, 2021, China | Prenatal yoga (32) | Routine prenatal care (32) | EPDS | 0.23 [-0.26; 0.72] | Some concerns |
| Sanaati et al,2016, Iran (Arm 2) | Lifestyle based education (62) | Routine prenatal care (63) | EPDS | 0.8 [0.45; 1.15] | Some concerns |
| Sanaeinasab et al, 2020, Iran | Spiritually integrated CBT (42) | Routine prenatal care (42) | DASS | 0.45 [0.02; 0.88] | High |
| Sanfillippo et al, 2020, Africa | Community psychosocial music intervention (39) | Routine prenatal care (60) | EPDS | 0.9 [0.49; 1.31] | Some concerns |
| Sapkota et al, 2020, Nepal | Psychosocial counselling intervention (70) | Routine prenatal care (70) | HADS | 0.62 [0.29; 0.95] | Some concerns |
| Su et al 2018, Taiwan | Omega 3 fatty acid supplementation (17) | Placebo- Olive oil capsule supplementation (16) | HAM-D | 0.98 [0.25; 1.71] | Low |
| Taniguchi and Sato, 2016, Japan | Physical activity intervention (54) | Routine prenatal care (43) | POMS | 0.19 [-0.18; 0.56] | Some concerns |
| Uebelacker et al, 2015, USA | Prenatal yoga programme (11) | Active control-Mum and baby wellness programme | QIDS | 0.47[-0.49; 1.43] | High |
| Van der Meulen et al, 2022, the Netherlands | Mindfulness based childbirth and parenting programme (75) | Enhanced usual care (66) | EPDS | 0.4 [0.07; 0.73] | Low |
| Vargas-Terrones et al, 2020, Spain | Exercise based intervention (36) | Routine prenatal care (25) | CES-D | 0.51 [0;1.02] | High |
| Vieten and Astin, 2008, USA | Mindfulness based intervention (13) | Routine prenatal care (18) | CES-D | 0.13 [-0.58; 0.84] | Some concerns |
| Vigod et al, 2019, Canada | Transcranial direct current stimulation (8) | Sham stimulation (9) | MADRS | 0.49 [-0.47; 1.45] | Low |
| Wahyuni et al, 2018, Pakistan | Spiritually based intervention (30) | Routine prenatal care (27) | EPDS | 0.69 [0.16; 1.22] | Some concerns |
| Wang et al, 2023, China | Mindfulness based childbirth and parenting programme (40) | Childbirth education course (43) | EPDS | 0.19 [-0.24; 0.62] | Low |
| Wirz-Justice et al, 2011, Switzerland | Bright light therapy (16) | Placebo- Dim light therapy (11) | SIGH-ADS-29 | 0.8 [0.02; 1.58] | Low |
| Woolhouse et al, 2014, Australia | Mindfulness based intervention (13) | Routine prenatal care (10) | CES-D | 0.3 [-0.52, 1.12) | High |
| Wulff et al, 2020, Germany (Arm 2) | Singing intervention programme (59) | Routine prenatal care (49) | EPDS | 0.13 [-0.24; 0.5] | High |
| Yang et al, 2019, China | Mindfulness intervention programme (62) | Routine prenatal care (61) | PHQ-9 | 0.93 [0.56; 1.3] | High |
| Yang et al, 2022, China (Arm 1) | Monitoring training mindfulness programme (37) | Emotion management training (34) | PHQ-9 | 0.14 [-0.31; 0.59] | Low |
| Yang et al, 2022, China (Arm 2) | Monitoring training with an emphasis on acceptance mindfulness programme (37) | Emotion management training (34) | PHQ-9 | 0.81 [0.32; 1.3] | Low |
| Yildirim et al, 2022, Turkey | Pilates based therapeutic exercise (17) | Routine prenatal care (17) | HADS | 1.06 [0.35; 1.77] | Low |
| Zenouzi et al,2021, Iran | Relaxation training programme (30) | Routine prenatal care (32) | DASS | 0.39 [-0.1; 0.88] | Some concerns |
| Zhang et al, 2018, China | Mindfulness Based Stress Reduction group programme (32) | Routine prenatal care (31) | SDS | 0.08 [-0.43; 0.59] | Some concerns |
| Zhang et al, 2023, Hong Kong | Mindfulness based childbirth and parenting programme (94) | Antenatal childbirth education and support (89) | EPDS | 0.83 [0.5; 1.16] | Low |
| Zhao et al, 2018, China | Group psychological intervention (176) | Routine prenatal care (176) | EPDS | 0.38 [0.18; 0.58] | Some concerns |
