# Supplementary material for: The evidence base of interventions to treat antenatal depression: a meta-analysis of randomized controlled trials
Source: Arch Womens Ment Health. 2026 Jul 3;29(4):103. doi: 10.1007/s00737-026-01723-0 (PMC13331926; doi:10.1007/s00737-026-01723-0)
Supplement: Supplementary file 2 — Supplementary Material 2 (DOCX 21.7 KB) [file 737_2026_1723_MOESM2_ESM.docx]

| **Author** | **Biomarker** | **Effect Size** | **Interpretation** | **Favours Intervention or Favours Control** |
| --- | --- | --- | --- | --- |
| Nishi et al (A) | Oestradiol | 0.24 | Previous studies have demonstrated low levels of oestradiol with clinical response to treatment in a perinatal population. Since T2 levels are lower in the treatment group, the intervention had a therapeutic effect (Nishi et al, 2019). | Favours intervention |
| Nishi et al (B) | hsCRP | 0 | The intervention had no impact on hsCRP levels. | N/A |
| Nishi et al (C) | IL-6 | 0.07 | Intervention group levels were lower at T2, indicating lower stress. | Favours intervention |
| Nishi et al (D) | Adiponectin | 0.26 |  | Favours intervention |
| Cao et al (A) | Systolic BP | 1.38 | Intervention group’s mean systolic BP was lower at T2. The music therapy intervention had a positive impact on blood pressure | Favours intervention |
| Cao et al (B) | Diastolic BP | 0.93 | Intervention group’s mean systolic BP was lower at T2. The music therapy intervention had a positive impact on blood pressure | Favours intervention |
| Cao et al (C) | Angiotensin II | 1.81 | The music therapy intervention was able to control Angiotensin II levels | Favours intervention |
| Sanaeinasab et al (A) | Systolic BP | 0.49 | Intervention group’s mean systolic BP was lower at T2 | Favours intervention |
| Sanaeinasab et al (B) | Diastolic BP | 0.34 | Intervention group’s mean systolic BP was lower at T2 | Favours intervention |
| Zhang and Emory (A) | Baseline cortisol | 0.29 | Intervention group’s baseline cortisol was lower at T2, indicating lower stress levels | Favours intervention |
| Zhang and Emory (B) | Reactive cortisol | 0.13 | Intervention group’s reactive cortisol was lower at T2, indicating lower stress levels | Favours intervention |
| Papandreou et al (A) | Glucose | 0.01 | Both groups have the same level of glucose at T2 | Marginally favours intervention |
| Papandreou et al (B) | Cholesterol | 0.7 | Intervention’s total cholesterol levels were lower than control at T2 | Favours intervention |
| Papandreou et al (B) | LDL | 0.27 | Intervention’s LDL levels were lower than control at T2. | Favours intervention |
| Wilczynska et al | Hair cortisol | -0.83 | Control group’s cortisol was lower than the intervention group at T2, indicating that the intervention had no therapeutic effect. | Favours control |
| Radmark et al (A) | A2M | -0.32 | Control group’s A2M levels were lower at T2 compared with the intervention group, indicating that the intervention had no therapeutic effect. | Favours control |
| Radmark et al (B) | Haptoglobin | -0.31 | Increased levels of haptoglobin indicate inflammation. As this was higher in the intervention group at T2 compared to control, the intervention did not have a therapeutic effect. | Favours control |
| Radmark et al (C) | SAP | -0.27 | Evidence suggests SAP levels are higher in those suffering from depression. (su et al, 2022)The intervention group’s T2 levels were higher than control, indicating that the intervention had no therapeutic effect. | Favours control |
| Radmark et al (D) | PCT | 0.45 | PCT levels in the intervention group were lower than the control group at T2, suggesting a therapeutic affect of the intervention. | Favours intervention |
| Radmark et al (E) | Ferritin | -0.28 | Evidence suggests that lower levels of ferritin are associated with postnatal depressive symptomology (O’Toole etal, 2023). Since ferritin levels in the intervention group were lower at T2, the intervention did not have a therapeutic effect. | Favours control |
| Radmark et al (F) | tPA | 0.07 | Research shows that higher tPA levels are associated with higher depressive symptoms (Tsai et al, 2017). Since T2 tPA levels are higher in the control group, the intervention had a marginal therapeutic effect. | Favours intervention |
| Radmark et al (G) | PCT | 0.45 | Research shows that higher PCT levels are associated with higher depressive symptoms (El Sayed et al, 2022). Since T2 PCT levels are higher in the control group, the intervention had a therapeutic effect. | Favours intervention |
| Radmark et al (H) | SAA | 0.48 | Research shows that higher SAAlevels are associated with higher depressive symptoms (El Su et al, 2022). Since T2 SAA levels are higher in the control group, the intervention had a therapeutic effect. | Favours intervention |
| Radmark et al (I) | Osteocalcin | -0.4 | Evidence has shown that patients with depression have lower osteocalcin concentrations (Skowrońska-Jóźwiak et al, 2020). Since T2 control group concentrations were lower than the intervention group, the intervention did not have a therapeutic effect. | Favours control |
