# Supplementary material for: The evidence base of interventions to treat antenatal depression: a meta-analysis of randomized controlled trials
Source: Arch Womens Ment Health. 2026 Jul 3;29(4):103. doi: 10.1007/s00737-026-01723-0 (PMC13331926; doi:10.1007/s00737-026-01723-0)
Supplement: Supplementary file 3 — Supplementary Material 3 (DOCX 23.5 KB) [file 737_2026_1723_MOESM3_ESM.docx]

| Income and Geographical Regions | K |
| --- | --- |
| 1. Geographical Regions |  |
| East Asia and Pacific | 28 |
| Middle East and North Africa | 24 |
| Europe and Central Asia | 30 |
| South Asia | 7 |
| North America | 22 |
| Latin America and Caribbean | 2 |
| Sub Saharan Africa | 2 |
| 1. Income Regions |  |
| High income | 59 |
| Upper middle income | 46 |
| Lower middle income | 9 |
| Low income | 1 |
