# Supplementary material for: The evidence base of interventions to treat antenatal depression: a meta-analysis of randomized controlled trials
Source: Arch Womens Ment Health. 2026 Jul 3;29(4):103. doi: 10.1007/s00737-026-01723-0 (PMC13331926; doi:10.1007/s00737-026-01723-0)
Supplement: Supplementary file 4 — Supplementary Material 4 (DOCX 18.2 KB) [file 737_2026_1723_MOESM4_ESM.docx]

| Analysis | SMD | 95% CI | *p* | *I^2^* | 95% CI |
| --- | --- | --- | --- | --- | --- |
| Main Analysis | 0.65 | 0.48- 0.83 | <0.0001 | 88% | 86%- 89.5% |
| Outliers Removed^1^ | 0.5 | 0.42-0.56 | <0.0001 | 46.3% | 31.5%-57.9% |
| ^1^Removed as outliers: "Zemestani and Nikoo", "Yazdanimehr et al", "Wulff et al (Arm 1)", "Shahtaheri et al", "Sanaati et al (Arm 1)", "Puertas-Gonzalez et al (Arm 2)", "Nishi et al", "Jabbari et al (Arm 1)", "Jabbari et al (Arm 2)", "Heller et al", "Field et al ( e)", "Akbarian et al", "Zhang and Emory", "Ussher et al", "Psaros et al", "Nejad et al", "Leung and Lam", "Kalmbach et al", "Daley et al", "Daley et al", "Kieffer et al", "Bose", "Wilczyńska et al.", "Zhang et al. (B)", "Hulsosch et al.", "Lewis et al.", "Lee et al.", "Lawson et al.", "Goma et al.", "Gennaro et al.", "Abujilban et al." | | | | | |
