# Supplementary figures and images for: The evidence base of interventions to treat antenatal depression: a meta-analysis of randomized controlled trials
Source: Arch Womens Ment Health. 2026 Jul 3;29(4):103. doi: 10.1007/s00737-026-01723-0 (PMC13331926; doi:10.1007/s00737-026-01723-0)

Figure 3a


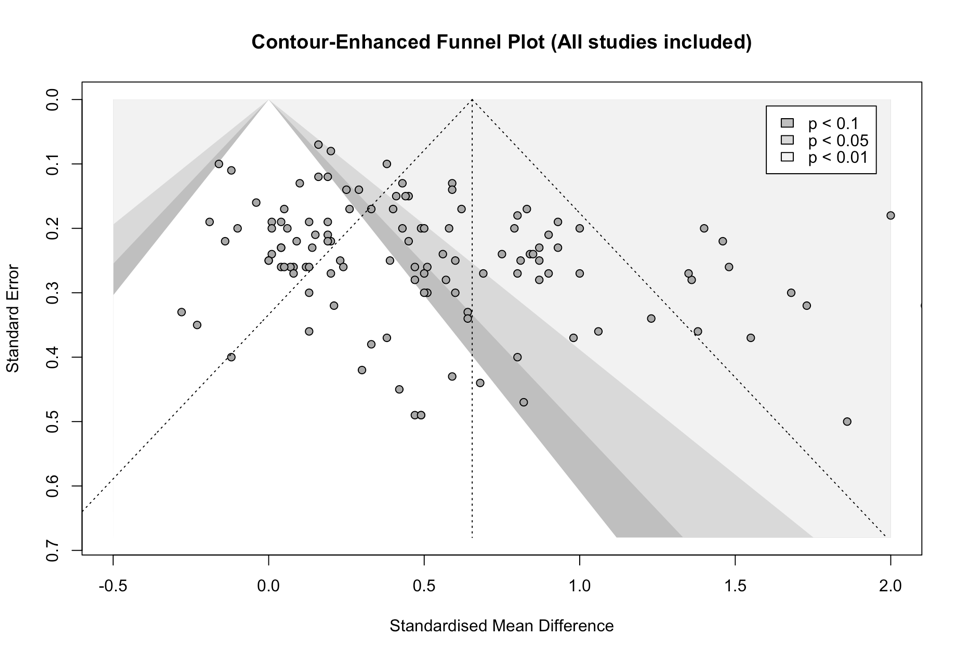


Figure 3b


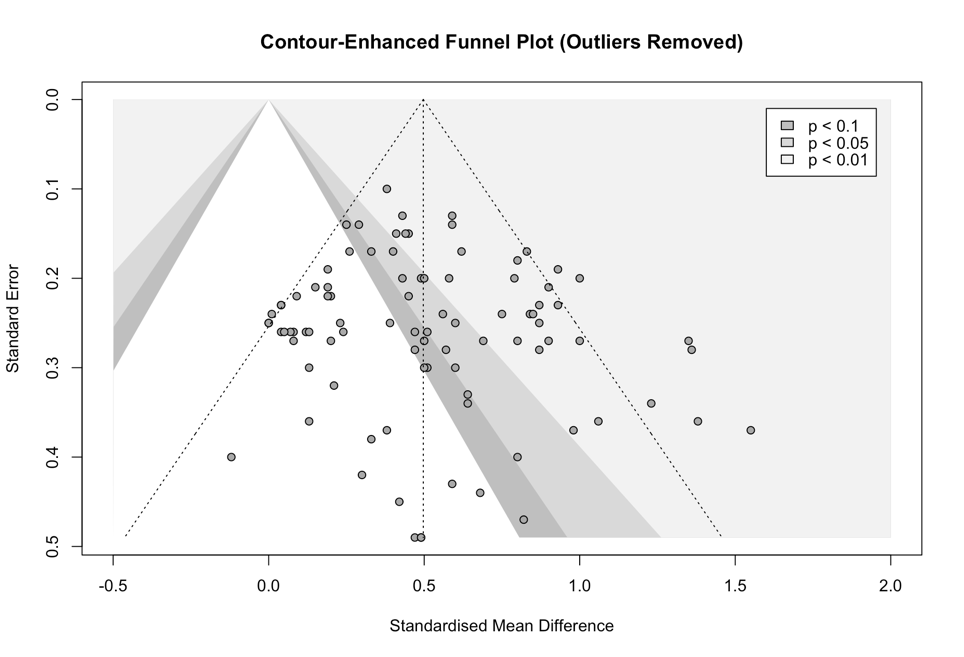

Supplement: Supplementary file 5 — Supplementary Material 5 (DOCX 218 KB) [file 737_2026_1723_MOESM5_ESM.docx]

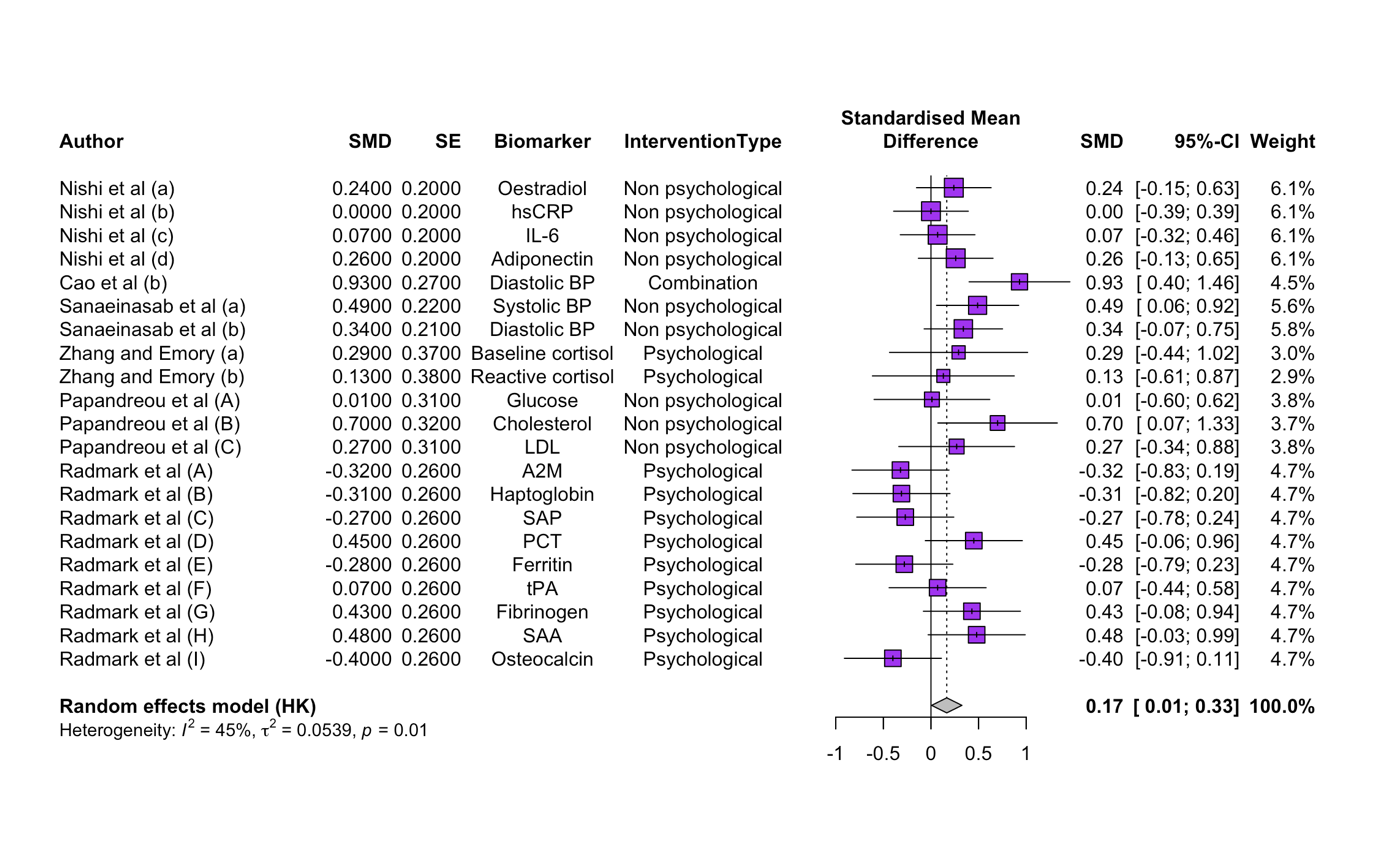

Supplement: Supplementary file 6 — Supplementary Material 6 (DOCX 364 KB) [file 737_2026_1723_MOESM6_ESM.docx]

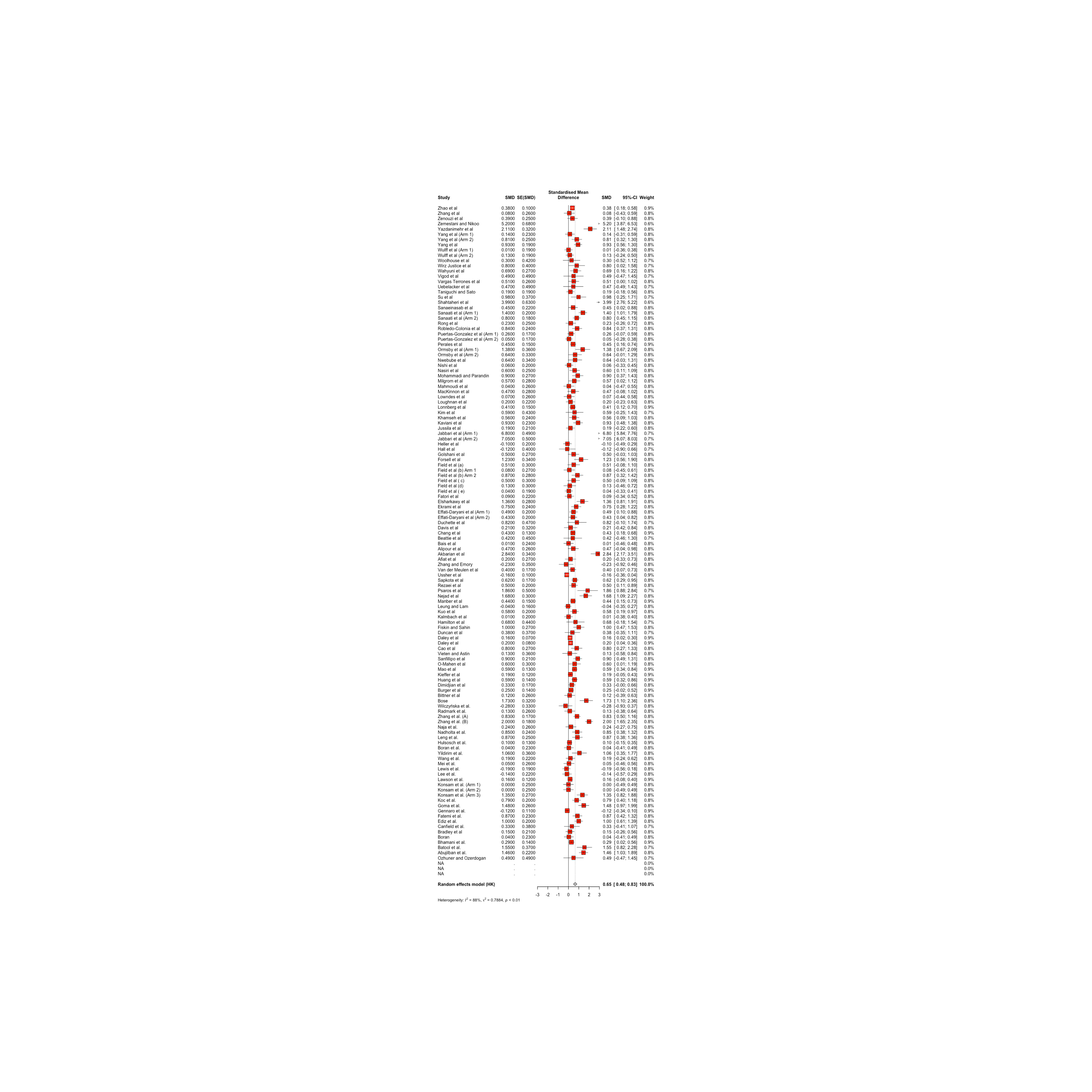

Supplement: Supplementary file 7 — Supplementary Material 7 (DOCX 1.03 MB) [file 737_2026_1723_MOESM7_ESM.docx]
